# Vascular plants and mosses as bioindicators of variability of the coastal pine forest (Empetro nigri-Pinetum)

**DOI:** 10.1038/s41598-023-50189-y

**Published:** 2024-01-02

**Authors:** Grzegorz J. Wolski, Zbigniew Sobisz, Józef Mitka, Andrzej Kruk, Ilona Jukonienė, Agnieszka Popiela

**Affiliations:** 1 Department of Geobotany and Plant Ecology, Faculty of Biology and Environmental Protection, University of Lodz, Banacha St. 12/16, 90-237 Łódź, Poland; 2 Institute of Biology and Earth Sciences, Pomeranian University, Arciszewskiego St. 22A, 76-200 Słupsk, Poland; 3 Faculty of Biology, Institute of Botany, Jagiellonian University, Gronostajowa St. 7, 30-387 Kraków, Poland; 4 Department of Ecology and Vertebrate Zoology, Faculty of Biology and Environmental Protection, University of Lodz, Banacha St. 12/16, 90-237 Łódź, Poland; 5 Nature Research Centre, Žaliųjų Ežerų St. 47, 12200 Vilnius, Lithuania; 6 Institute of Biology, University of Szczecin, Felczaka St. 3C, 71-412 Szczecin, Poland

**Keywords:** Biodiversity, Community ecology, Ecosystem ecology, Forest ecology

## Abstract

Empetro nigri-Pinetum is a unique sea coast plant community developing along the Baltic Sea from Germany to Lithuania. Our detailed field research of bryophytes and vascular plants has highlighted the regional diversity of the Empetro nigri-Pinetum typicum plant community throughout its range in Central Europe. Our study indicated that vascular plants and mosses effectively discriminate against the described phytocoenoses, thus it was possible to distinguish three variants of the coastal forest: *Calluna–Deschampsia* (from Germany), *Vaccinium vitis–idaea* (from Poland) and *Melampyrum–Deschampsia* (from Lithuania). Redundancy analysis indicated that the division is related to the habitat conditions of the analyzed areas, with humidity having the greatest impact on this differentiation. Kohonen’s artificial neural network (i.e. self-organising map, SOM) confirmed the heterogeneous nature of the studied phytocenoses, and combined with the IndVal index enabled identification of indicator species for respective studied patches: *Deschampsia flexuosa* for *Calluna*–*Deschampsia* group; *Aulacomnium palustre*, *Calluna vulgaris*, *Carex nigra*, *Dicranum polysetum*, *Erica tetralix*, *Oxycoccus palustris*, *Sphagnum capillifolium*, *Vaccinium uliginosum* and *Vaccinium vitis*–*idaea* for *Vaccinium vitis*–*idaea* group; and young specimens of *Betula pendula*, *Lycopodium annotinum*, *Melampyrum pratense* and *Orthilia secunda* for *Melampyrum*–*Deschampsia* group. Thereby, our study showed that individual groups of species can be very good bioindicators for each of the studied phytocoenoses.

## Introduction

In Central Europe, plant communities are studied in accordance with the commonly-accepted method given by the Central European School of phytosociology (Zürich-Montpellier, CEPF^1^). Despite Braun-Blanquet approach is relying on floristic composition of vascular plants and species similarity, cryptogams are often underestimated or omitted during such procedures, and are hence incompletely represented in syntaxonomic systems^2–6^. Among these, a commonly overlooked group of cryptogams are the bryophytes, i.e. the mosses, liverworts and hornworts. This fact is surprising, especially taking into account our knowledge of the outstanding bioindicative value of bryophytes, both in terms of the substrate (including soil, wood, trees or rocks) they cover and phytocoenoses where they grow^7–10^.

Hence, it seems reasonable to examine the role of bryophytes in plant communities more deeply. Such study approach would allow the structure of plant communities to be fully documented. Additionally, more advanced statistical methods may enable the identification of groups of indicator species, both in terms of quality and quantity. It would be particularly interesting to determine the extent to which the value of indicator species may differ from that currently accepted in the phytosociological literature e.g.^3,5,10,11^.

Among the forest communities, a model group for such an analysis is the coastal pine forest Empetro nigri-Pinetum. It is a permanent community that ends its succession among sea sand dunes, and is well distinguished among other pine forests in Europe. It is characterized by a specific physiognomy due to the special habit of pines and the almost constant presence of *Empetrum nigrum* ssp. *nigrum* in the undergrowth^12^. Its range covers the South Baltic Coast, in a narrow strip of coastal dunes stretching across Germany, Poland and Lithuania^12–14^. The community was selected for the present study due to its relatively wide range (about 780 km of the Baltic coast), small patches, good state of preservation in protected areas, and its large share of bryophytes.

Thus, the aim of the research was to perform a detailed qualitative and quantitative analysis of Empetro nigri-Pinetum typicum throughout its range in Europe with reference to the habitat and climate conditions. Wherein, the analyzed qualitative feature was the species (vascular plants and bryophytes) composition, and the quantitative one was their abundance.

Several working goals were adopted in the study: analysis of the species composition of the Empetro nigri-Pinetum typicum phytocoenosis; assessment of the frequency of occurrence and abundance of individual taxa; analysis of the similarity of the examined phytocenoses; analysis of results in the context of habitat and climatic conditions and assessment of bioindicative values of individual taxa.

The conducted research was based on four hypotheses: (H1) the main driver of Empetro-nigri Pinetum heterogeneity are climate differences; (H2) there are indicator species differentiating individual patches, and (H3) bryophytes have a higher indicative potential than vascular plants.

## Materials and methods

### Selection of the sampling sites

**Figure 1:**
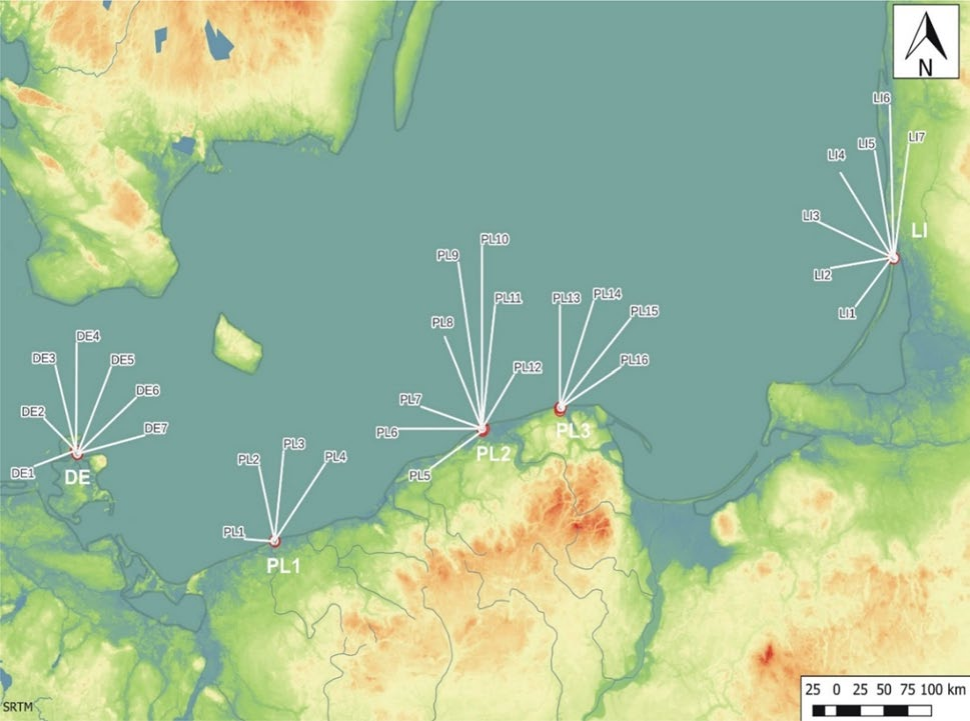
Location of the five test points (DE in Germany, PL1–PL3 in Poland, LI in Lithuania) and thirty (DE1–DE7, PL1–PL16, LI1–LI7) studied plots in Central Europe.

Thirty study plots were established at five test points (DE, PL1, PL2, PL3, LI) within the entire range of Empetro nigri-Pinetum typicum in Europe. This stretches across Germany (DE), Poland (PL) and Lithuania (LI). Each plot measured 20 m × 20 m (Fig. 1).

In order for the studied patches to be preserved as well as possible and not transformed, it was assumed that only phytocoenoses located within the borders of protected areas, i.e. national parks and nature reserves, would be selected for research. The number of plots in each country was adjusted to the area occupied by the described phytocoenosis in that country. Thus, in Germany and Lithuania, seven (in each country) plots were established—in German (DE1–DE7) and Lithuania (LI1–LI7), while in Poland, 16 plots were established (PL1–PL16) (Fig. 1).

The results of the grouping analysis showed that some study plots of the examined areas are very similar to each other; therefore, in subsequent analyses test points PL2 and PL3 were treated as one area and marked with a symbol PL23 (Fig. 1).

In addition, only patches with the presence of *Empetrum nigrum* were selected for the study, as this was the only species distinguishing the coastal forest from the pine forest.

### Vegetation data

The material was acquired from studies carried out using a modified phytosociological method. First, all tree species in the basic study area (400 m^2^) were recorded. The number of individuals was recorded for each species. Then, on the same plot, but on a smaller area (100 m^2^), a phytosociological *relevé* was performed using the classic Braun-Blanquet^1^ method (RawDate1).

In order to determine the density of individuals of vascular plants within the examined area of 100 m^2^, 25 randomly-located test plots with an area of 1/6 m^2^ were designated. Within these plots, all individual plants were counted. In the case of plants such as grasses or sedges, a single clump of a given species was considered a single occurrence.

In the case of bryophytes, only epigeic species recorded throughout the entire study area (100 m^2^) were included. Each separate turf of a given species, with an area of not more than 20 cm × 20 cm, at a distance of at least 20 cm from another turf of the same taxon, was treated as a separate record. In order to assess the abundance of species with more extensive turf (e.g. *Pleurozium schreberi* or *Dicranum scoparium*), the number of squares (20 cm × 20 cm) contained in the examined patch of mosses was assessed^10^.

Climate data was obtained from a free database https://pl.climate-data.org/ (RawDate2). Field research was carried out in the summer of 2022 on the basis of the following permits: in Poland decision of the Minister of Climate and Environment No. DOP-WOPPN.61.85.2022.MŚP; decision No. RDOŚ-Gd-WOC.6205.54.2022.MaK.3 of the Regional Directorate for Environmental Protection (RDEP) in Gdańsk; decision No. WOPN.6205.12.2022.AS of RDEP in Szczecin. Research in other areas (outside Poland) did not require any permits to enter and/or collect plants. The map of the study location was made on the basis of the numerical terrain model-Shuttle Radar Topography Mission (SRTM), in the QuantumGIS software version 3.26.3.

The names of vascular plants and bryophyte species are given after^15–17^.

### Data analyses

The structure of species dominance in a given area was determined by dividing the number of occurrences of a given species by the total number of individuals in a given area (expressed in %). The PERMANOVA was used to determine the significance of differences among the areas. In the analyzes of cryptogams, sporadic specimens (total number of occurrences < 20) were omitted in numerical analyses. They were later included in the overall analyzes of the flora.

Redundancy analysis (RDA) was used to determine the relationship between species abundance, location and environmental variables (mean annual: temperature, precipitation, humidity, rain days, number of sunny hours per year) (RadDate5). Principal component analysis (PCA) was used to determine the relationship between species abundance and location. A multiple regression via the generalized linear model template (GLM) with the Poisson response distribution was used to display the species scores along the first two PCA axes; a Poisson distribution for the random component is the simplest GLMs for counting data. The significance of the response (I-type error) was tested with parametric tests based on a partial F statistic.

Redundancy analysis (RDA) was performed with the relevé groups as explanatory variables. Random Monte Carlo permutations (n = 499) were carried out to display the significance of the particular species using the conditional term effects. Also, the significance of the groups delimited was checked with random permutations and simple term effects. PCA and RDA were carried out with Canoco for Windows^18^. In order to determine the statistical significance of groups of phytosociological records (temporarily referred to as "syntaxa"), extracted on the basis of numerical classification, the Random Forest algorithm was used^19^. This allowed an evaluation of the accuracy of assigning objects to distinguished classes (syntaxa). The Gini coefficient was calculated to indicate the unevenness of the distribution of features in groups. High index values indicate a high classification value. To calculate the Gini indicator, the "randomForest" set and the importance procedure were used, as well as their graphical presentation in the varImpPlot program in the R software package^19^. For the the above-mentioned analysis, boxplots were created using the boxplot *function*, the nonparametric Kruskal–Wallis by rank test was performed using the *kruskal.test* function, whereas the function *pairwise.wilcox.test* was used to calculate pairwise comparisons among group levels with BH testing corrections^10^.

The extent to which the variance of the forest stands was explained by the vascular plants and bryophyte species and their interactions was then evaluated. Briefly, a variation partitioning procedure based on RDA, was carried out with *rdacca.hp*^20^, and a diagram was produced with *draw.pairwise.venn* in “veneuler” R Package^19^.

The patterns in the plots were recognised with a self-organizing map (SOM^21,22^), also referred to as Kohonen’s (unsupervised) artificial neural network (ANN). ANNs do not require a priori specification of the model underlying a studied phenomenon because they learn it based on the processed data. This ability also applies to variables related in a non-linear way or exhibiting non-normal distributions^23,24^, both typical for environmental studies.

ANNs are built of processing units called artificial neurons. In Kohonen’s ANNs they are arranged in two (input and output) interconnected layers. The input layer serves for data input, and an output layer is responsible for data structuring and output. The data set used in this study (log-transformed abundances of 43 taxa × 30 plots) was displayed on the input layer of 43 neurons (one input neuron per taxon). A total of 24 output neurons were arranged as a 6 × 4 rectangular lattice. During the network training process, each input neuron repeatedly, i.e. eight times in the rough training phase and 32 times in the fine-tuning phase, transmitted signals of modified weight to all the output neurons. On this basis, a virtual plot was created in each output neuron. The input neurons had no further significance in pattern recognition^25,26^.

The distance between virtual plots (and respective output neurons) reflected their mutual dissimilarity, i.e., virtual plots in distant neurons were different while those in neighbouring neurons were similar. That was not true when the neighbouring neurons belonged to different (sub)clusters. The virtual plots (and respective output neurons) were clustered by hierarchical cluster analysis based on the Ward algorithm and Euclidean distance measure^27,28^. When a given virtual plot matched all real plots less than any other virtual plot, the respective output neuron remained empty. Conversely, when a given virtual plot was the best match for more than one real plot, the respective output neuron was assigned several real plots. In this way, finally all the real plots became assigned to output neurons in such a way that similar real plots were in the same neuron or in neighbouring ones, and considerably different real plots were in distant neurons^29,30^.

For network training process the batch training algorithm was chosen because its results are independent of the order of the input variables, and because it does not require a training rate factor to be specified^31^. The training and clustering processes were carried out using the SOM Toolbox^28^ (http://www.cis.hut.fi/projects/somtoolbox/).

The SOM Toolbox also allows the associations between vascular plants or bryophyte species in virtual plots and the respective output neurons to be visualised in the form of greyness gradients^26^. However, it does not provide any statistical verification of those associations. For this purpose the untransformed taxa abundances were subjected to the Indicator Species Analysis (ISA)^32^. ISA identifies the taxa that are significantly associated with each (sub)cluster of real plots, and hence respective environmental conditions.

ISA is based on indicator values (IndVals):

$${\text{IndVal}}_{ij} = {\text{ A}}_{ij} \times {\text{ F}}_{ij} \times { 1}00$$

A_*ij*_ is the abundance_*ij*_/abundance_*i.*_. F_*ij*_ is the N plots_*ij*_/N plots_*.j*_where: A_*ij*_ is the mean abundance of taxon *i* in the plots of (sub)cluster *j* divided by the sum of average abundances of the taxon *i* in all the (sub)clusters, F_*ij*_ is the the relative frequency of occurrence of taxon *i* in the plots of (sub)cluster *j*, constant 100 is the applied in order to obtain the percentages.

Significant IndVals (and therefore indicator taxa) for (sub)clusters were identified with the Monte Carlo randomisation test. The IndVals were calculated and tested with PC-ORD statistical software^33^.

Species richness was compared between the SOM subclusters with the Kruskal–Wallis test and the post hoc Dunn test^34,35^.

## Results

### General results

During the research, 39 taxa, including 17 bryophytes species (one liverwort and 16 mosses), 21 vascular plants and one lichen (*Cladonia* sp.) were recorded (RawDate1, 3). The phytocoenosis that is the subject of our research should be classified as the Empetro nigri-Pinetum typicum association in three varieties reflected in the geographical distribution from west to east: *Calluna–Deschampsia* (RawDate1, plots DE1–DE7), *Vaccinium vitis–idaea* (RawDate1, plots PL1–PL16) and the varieties *Melampyrum–Deschampsia* (RawDate1, plots LI1–LI7).

The *Vaccinium vitis–idaea* variety occurring on the Polish coast is the richest in species. Here it is possible to distinguish a variety from wet habitats (RawDate1, plots 10–14), which is indicated by the presence of *Vaccinium uliginosum*, *Erica tetralix*, *Andromeda polifolia*, *Oxycoccus palustris* as well as *Sphagnum capillifolium* and *Aulacomnium palustre*. Plots 17–19 (RawDate1) document the transition of patches to higher trophic communities, and transformed phytocoenoses. The *Vaccinium myrtillus* reach their optimum here. *Deschampsia flexuosa*, young pine and beech trees and the invasive species of moss *Orthodontium lineare* are also present.

The fragments of the discussed community, i.e. the variety of *Calluna-Deschampsia* developing on Rügen, are poorer in terms of species: the mean number of taxa in the plots is 10, however, the presence of massively occurring grass gives a characteristic accent to the physiognomy; noteworthy is the smaller number of bryophyte species (average number of taxa 5) and the lack of *Dicranum polysetum* moss, common in other locations (RawDate1).

The variety of *Melampyrum-Deschampsia* from the Curonian Spit (RawDate1, plots 24–30) refers to transitional forest communities in the direction of Betulo-Quercetum. This is evidenced by the presence of *Pinus* and *Sorbus* juveniles, as well as *Trientalis europea*, *Vaccinium myrtillus* and *Deschampsia flexuosa*. In the the studied phytocoenosis, the most important role is played by four species of mosses, found in each patch: *Pseudoscleropodium purum*, *Pleurozium schreberii*, *Hylocomnium splendes* and *Dicranum polysetum*. The most numerous is *Pseudoscleropodium purum*, which forms compact, extensive turfs (RawDate1).

### Bryophytes

In the entire study area, the following bryophytes were most often noted: *Pseudoscleropodium purum* (1323 records), *Pleurozium schreberi* (587 records) and *Dicranum polysetum* (340 records). The least frequent were e.g. *Brachythecium salebrosum*, *Pohlia nutans* and *Polytrichum juniperinum* (one records each) (Table 1, RawDate3).

**Table 1:** Mean numbers of the most common moss species and their percentage presence in the study area.

Species	Test points			
	DE (%)	PL1 (%)	PL23 (%)	LI (%)
*Pseudoscleropodium purum*	45 (53)	38 (48)	43 (40)	49 (47)
*Pleurozium schreberi*	18 (21)	12 (14)	21 (20)	24 (23)
*Dicranum polysetum*	1 (0.2)	11 (13)	17 (16)	13 (12)
*Hypnum jutlandicum*	14 (16)	10 (12)	6 (5)	4 (4)
*Dicranum scoparium*	4 (5)	5 (6)	6 (5)	5 (5)
*Hylocomium splendens*	3 (4)	5 (6)	12 (11)	9 (9)
*Sphagnum capillifolium*	0 (0)	0 (0)	2 (2)	0 (0)

Despite the similarities related to the dominant species, the test points are quite distinct in terms of quality and quantity of bryophyte taxa recorded on their surfaces (RawDate1). These observations are also confirmed by the analysis of biodiversity indicators (Simpson 1-D and Shannon H) (Supplementary Table 1).

The PERMANOVA analysis based on the bryophyte species of the studied areas found statistically significant differences (p < 0.05) between DE and PL23 and between DE and LI, while the PL1 area was not significantly different to other study areas. The analysis also showed that in terms of bryophytes, there are no statistically significant differences between the PL23 and LI areas (Supplementary Table 2).

**Figure 2:**
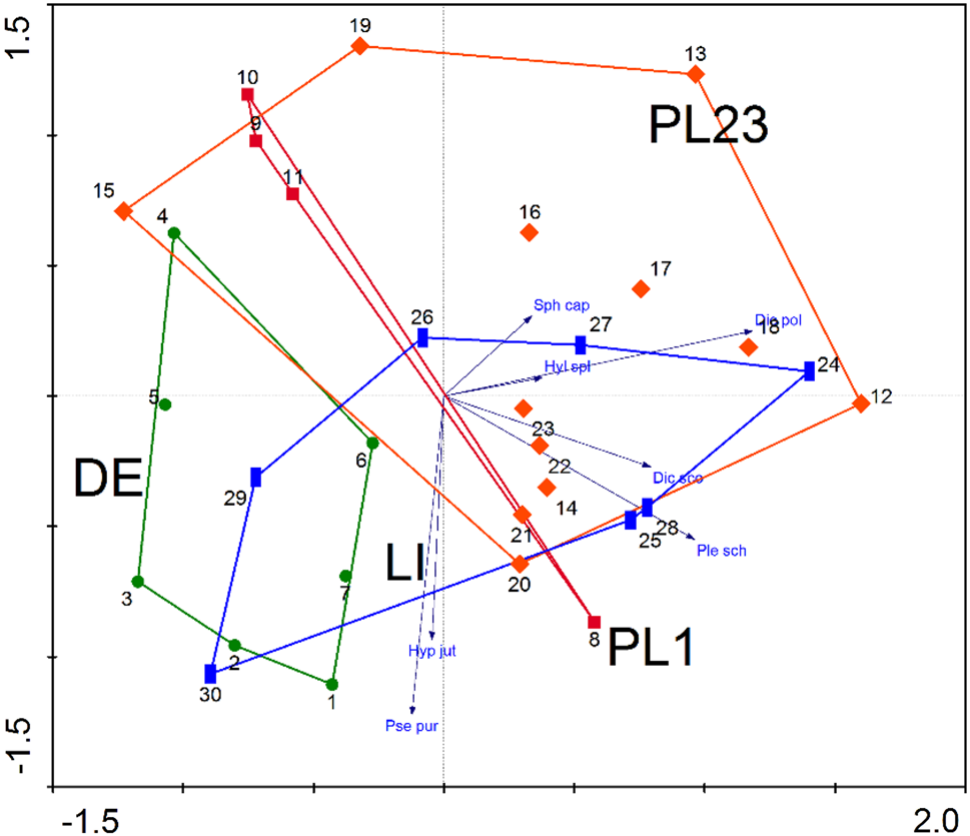
PCA analysis based on bryological data of individual test points. Explanation: DE, PL1, PL23 and LI refer to the areas mentioned in the Materials and methods and shown in Fig. 1.

The PCA analysis, which included abundances of bryophytes recorded in individual areas, showed that both axes explained 60% of the total variation (Fig. 2). It also showed that the groups of bryophyte species of individual areas quite clearly overlap; only the group from Germany (DE area) partly differs from the other groups (Fig. 2).

**Figure 3:**
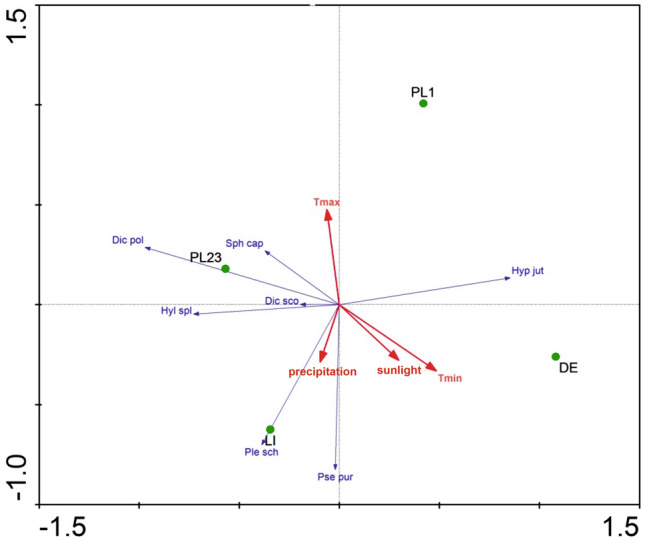
The results of the RDA analysis for the bryological data of the test points and the analyzed climatic variables. Explanation: DE, PL1, PL23 and LI refer to the areas mentioned in the Materials and methods and shown in Fig. 1. Tmin—minimum temperature, Tmax—maximum temperature.

The results of the RDA (Fig. 3, RawDate5) analysis indicate that the number of records of *Pseudoscleropodium purum* is most affected by humidity, compared to other environmental factors, with the two variables being positively correlated; thus, *P*. *purum* is most common in Lithuania (LI test point), and rarest in Poland (PL1). In contrast, the increase in temperature has practically no effect on the number of its records. Our findings also indicate that increases in humidity had the greatest impact on the number of records of *Pleurozium schreberi*, but not on the number of *Dicranum polysetum*, *D*. *scoparium* or *Hylocomium splendens*; the latter three species are the most common in Poland (PL23 test point).

### Vascular plants

During the research, 21 species of vascular plants were recorded in the entire transect of the studied phytocoenoses Empetro nigri-Pinetum typicum (RawDate3). Among the vascular plants, the following were most frequently recorded: *Empetrum nigrum* (7286 records), *Deschampsia flexuosa* (3153) and *Vaccinium vitis*–*idaea* (3065 records). On the other hand, the least frequently recorded were *Myrica gale* (one record), *Veronica chamaedrys* (two) and *Sorbus aucuparia* (three records) (RawDate3).

The study areas differ significantly in terms of recorded species of vascular plants. However, despite noticeable differences, *Empetrum nigrum* was recorded everywhere among the dominants; remaining dominants were *Deschampsia flexuosa*, *Vaccinium myrtillus*, *V*. *vitis*–*idaea* and *Calluna vulgaris* (Table 2), depending on the location of the test points.

**Table 2:** Mean numbers of the most common vascular plants and their percentage presence in the study areas.

Species	Test points			
	DE (%)	PL1 (%)	PL23 (%)	LI (%)
*Empetrum nigrum*	260 (35)	192 (30)	202 (32)	325 (62)
*Deschampsia flexuosa*	354 (48)	33 (5)	0 (0)	78 (15)
*Vaccinium myrtillus*	49 (7)	194 (30)	10 (3)	7 (1)
*Vaccinium vitis-idaea*	0 (0)	69 (11)	232 (37)	0 (0)
*Calluna vulgaris*	47 (6)	75 (12)	111 (18)	0 (0)
*Pinus sylvestris*	32 (4)	32 (5)	25 (4)	22 (4)
*Melampyrum pratense*	0 (0)	42 (7)	19 (3)	53 (10)
*Lycopodium annotinum*	0 (0)	0 (0)	0 (0)	19 (4)
*Carex nigra*	0 (0)	0 (0)	17 (3)	0 (0)
*Trientalis europaea*	0 (0)	0 (0)	0 (0)	13 (3)
*Erica tetralix*	0 (0)	0 (0)	9 (1)	0 (0)
*Orthilia secunda*	0 (0)	0 (0)	0 (0)	8 (2)
*Oxycoccvus palustris*	0 (0)	0 (0)	3 (0)	0 (0)
*Vaccinium uliginosum*	0 (0)	0 (0)	3 (0)	0 (0)
*Andromeda polifolia*	0 (0)	0 (0)	1 (0)	0 (0)

During the research, the largest number of vascular plants was recorded in Poland (PL23 and PL1 test points) and Lithuania (LI), while the least were recorded in Germany (DE test point). These observations are also confirmed by the analysis of biodiversity indicators (Simpson 1-D and Shannon H) (Supplementary Table 3).

**Figure 4:**
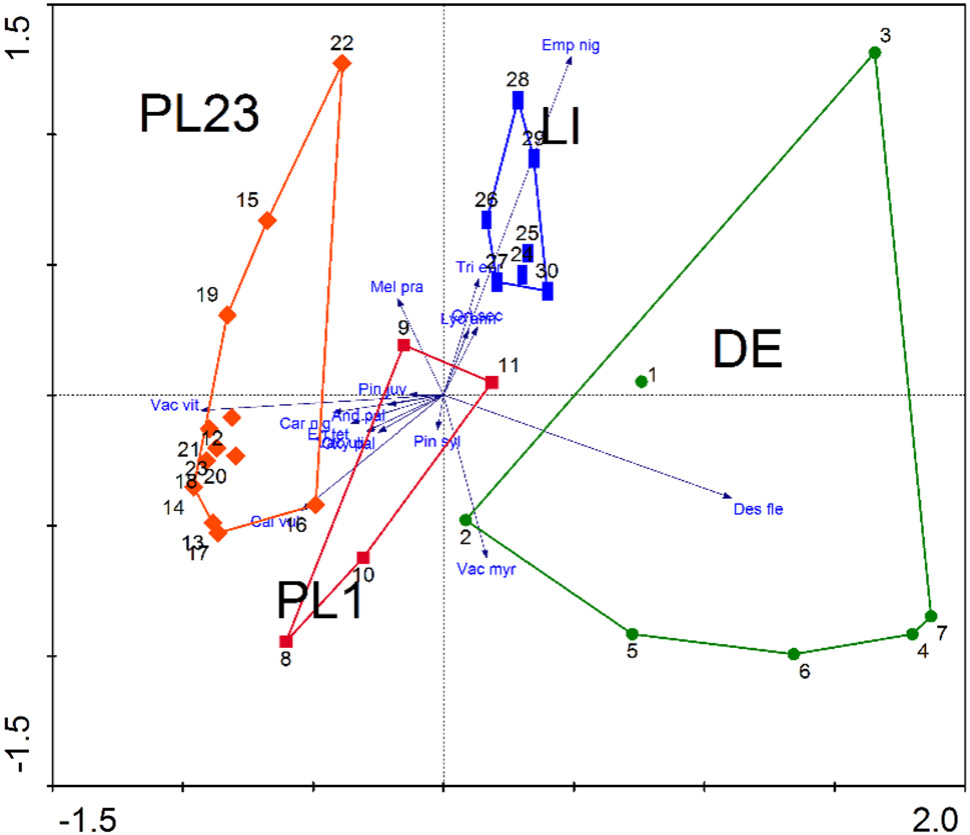
PCA analysis for vascular plants performed for individual test points.

The PERMANOVA analysis based on the abundance of individual species in the study areas showed that statistically significant differences (p < 0.05) occur between all study areas (Supplementary Table 4). The PCA analysis, which included the abundance of vascular plants recorded in individual areas, showed that the two axes together explain 70% of the variability: PC1 explains 50%, while PC2 explains 20% (Fig. 4). In addition, the PCA clearly shows that the species of individual areas form separate groups (Fig. 4).

**Figure 5:**
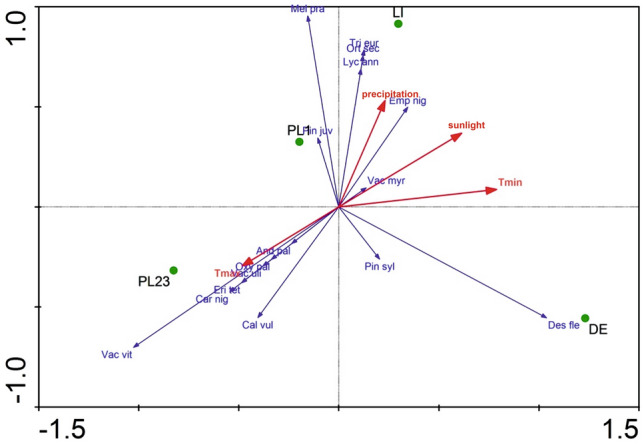
The results of the RDA analysis for the vascular plant data of the test points and the analyzed climatic variables. Explanation: DE, PL1, PL23 and LI refer to the areas mentioned in the Materials and methods and shown in Fig. 1. Tmin—minimum temperature, Tmax—maximum temperature.

The RDA analysis (RadDate5) showed that the first axis explains 71% of the variability in the canonical part (42.5% of the total) while the second explains 18% (10%) (Fig. 5). Also, the number of records of *Empetrum nigrum* is most affected by humidity, being most common in Lithuania (LI) and rarest in Poland (PL23 test point). However, an increase in temperature does not affect the number of records. It also appears that the increase in humidity has a strong impact on the number of occurrences, e.g. the number of sites of *Vaccinium uliginosum*, *Erica tetralix* and *Vaccinium vitis–idaea* decreases as humidity increases. These species are most frequently recorded in Poland (PL23) and least frequently in Lithuania (LI test point) (Fig. 5).

### Analysis of the entire flora of the Empetro nigri-Pinetum phytocoenosis

**Figure 6:**
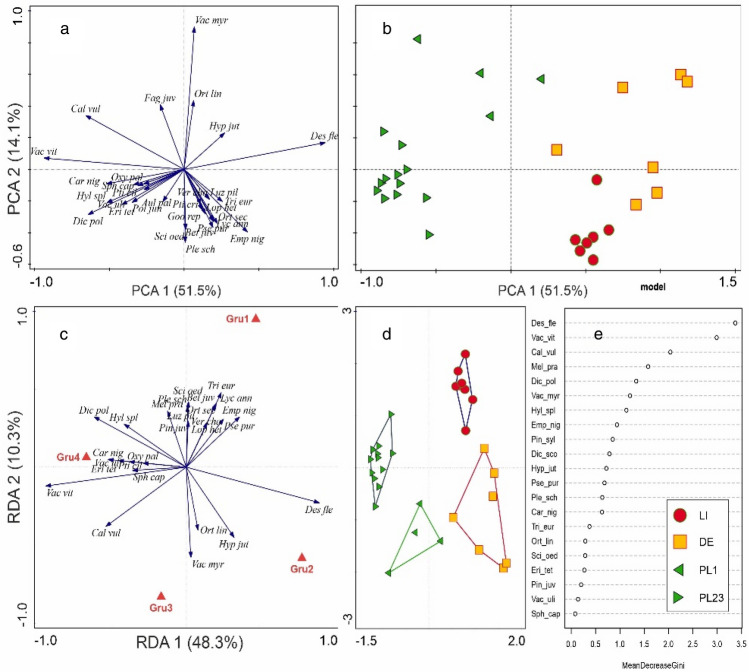
PCA i RDA of the studied flora Empetro nigri-Pinetum association in the Baltic region: (**a**) species, (**b**) plots. RDA: (**c**) species, (**d**) plots; (**e**) Gini index; DE (Germany), LI (Lithuania), PL1 and PL 23 (Poland test points).

The PCA ordination of plots enabled four groups of plant communities to be distinguished (Fig. 6b). Along Axis 1, PL1 has an intermediate position between PL23 (left side of the diagram), and DE and LI (right side).

The distribution of groups of plots is related to their species distribution (Fig. 6a). Axis 1 is loaded negatively with *Carex nigra* group, *Vaccinium vitis–idaea* and *Calluna vulgaris* and positively with *Deschampsia flexuosa* and *Empetrum nigrum* group. Axis 2 is loaded positively with *Vaccinium myrtillus*.

The RDA ordination with the explanatory variable “groups” clearly delimited the syntaxa and groups of species (Fig. 6c, d). The first axis explained 48.3% of the total variability and the second 10.3%. The gradient along the axis 1 is described by *Vaccinium vitis–idaea* and related species, and in the opposite position, by *Deschampsia flexuosa*. The gradient along axis 2 concerns *Vaccinium myrtillus*, *Orthodintium lineare* and *Hypnum jutlandicum* and, at the opposite end, by a group of species with *Pleurozium schreberi*. This pattern corresponds to the distribution of the plots and species in PCA.

**Figure 7:**
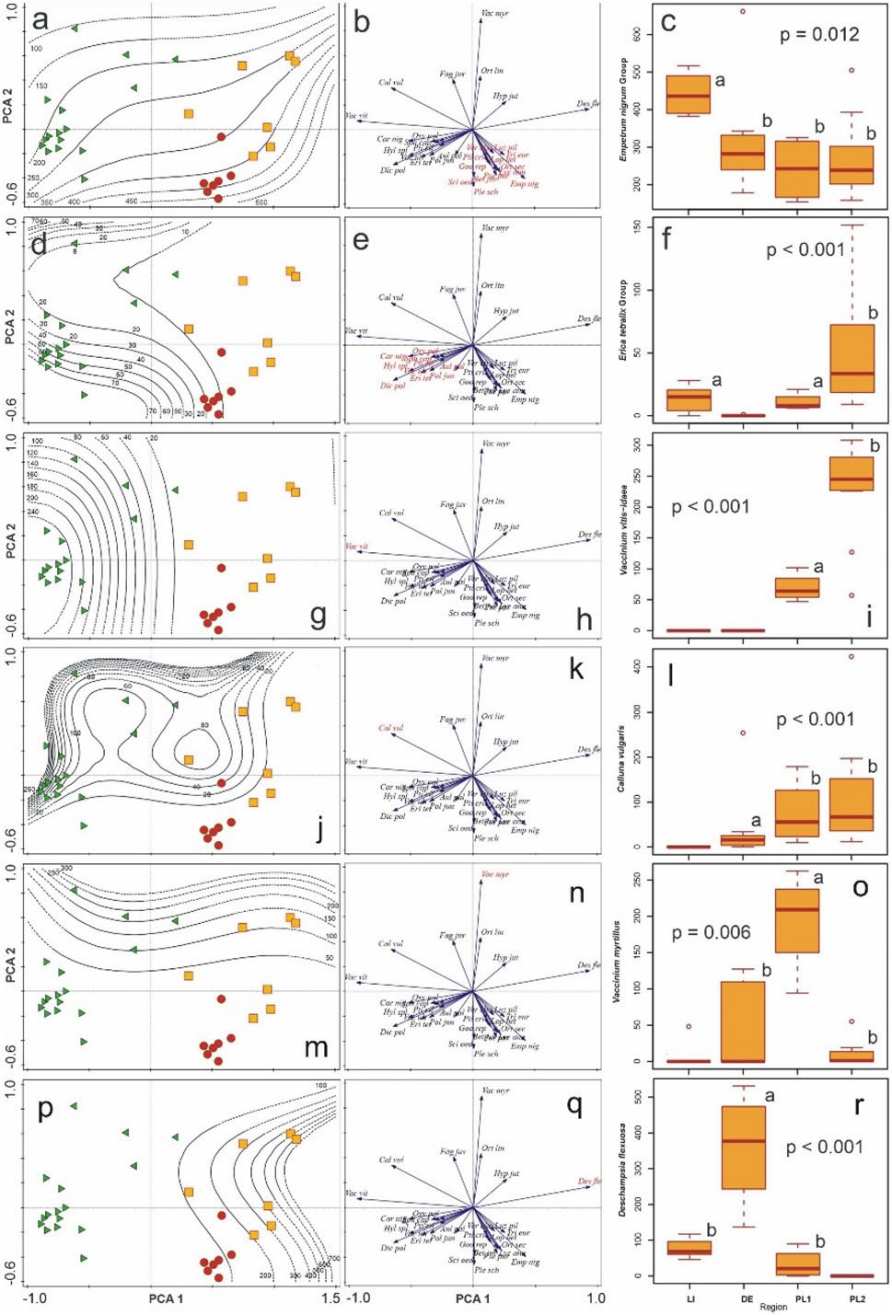
GLMs, PCA (with the relevant species or species groups in red) and boxplots for the species or species groups responsible for Empetro nigri-Pinetum variability in the Baltic region. All regressions are highly significant (p < 0.001). The boxplots show I-type errors of the Kruskal–Wallis test. Statistically significant differences among means based on posteriori tests (p < 0.05); in contrast, the presence of the same letters indicates a lack of statistical significance (p > 0.05). (a–c) *Empetrum nigrum* group; (**d–f**) *Carex nigra* group; (**g–i**) *Vaccinium vitis–idaea*; (**j–l**) *Calluna vulgaris*; (**m–o**) *Vaccinium myrtillus*; (**p–r**) *Deschampsia flexuosa*. For vegetation sites LI, DE, PL1 and PL23 see Fig. 6.

The distribution of the species (or species groups) scores in the generalized linear model (GLM), and their share in particular plant communities, is shown in Fig. 7.

The Gini index (Fig. 6e) shows the group of species with the highest discrimination value: *Deschampsia flexuosa*, *Vaccinium vitis–idaea*, *Calluna vulgaris*, *Melampyrum pratense*, *Dicranum polysetum*, *Vaccinium myrtillus* and *Hylocomium splendens*.

The distinguished four plant communities are characterized by different shares of the particular species groups. The *Empetrum nigrum* group dominates in LI, while *Deschampsia flexuosa* has the highest counts in DE (Germany). The highest share of *Vaccinium myrtillus* is found in PL1, a part of the Polish material. All Polish test points (PL1 and PL23) also have high counts of *Calluna vulgaris* and *Vaccinium vitis–idaea*. Polish test point PL23 has the highest count of *Carex nigra* group and a null count of *Deschampsia flexuosa*. The significance of particular species was evaluated using the permutation test (Supplementary Table 5).

In the *Empetrum nigrum* group, none of the species demsontrated statistical significance. In the *Erica tetralix* group, *Oxycoccus palustris* and *Dicranum polysetum* were significant (p = 0.042), and *Erica tetralix* nearly significant (p = 0.09). The “other” group generally had significant (p = 0.04) or nearly significant (p = 0.06–0.07) I-type error values.

Variance partitioning highlighted the importance of bryophyte species in explaining the variability of the coastal forest. Bryophytes explained 15% of the total variability and vascular plant species 39%, and together they explained 22%. In the effect, partitioning indicted that 62% was explained by vascular plants, and 38% by bryophytes (Supplementary Fig. 1).

**Figure 8:**
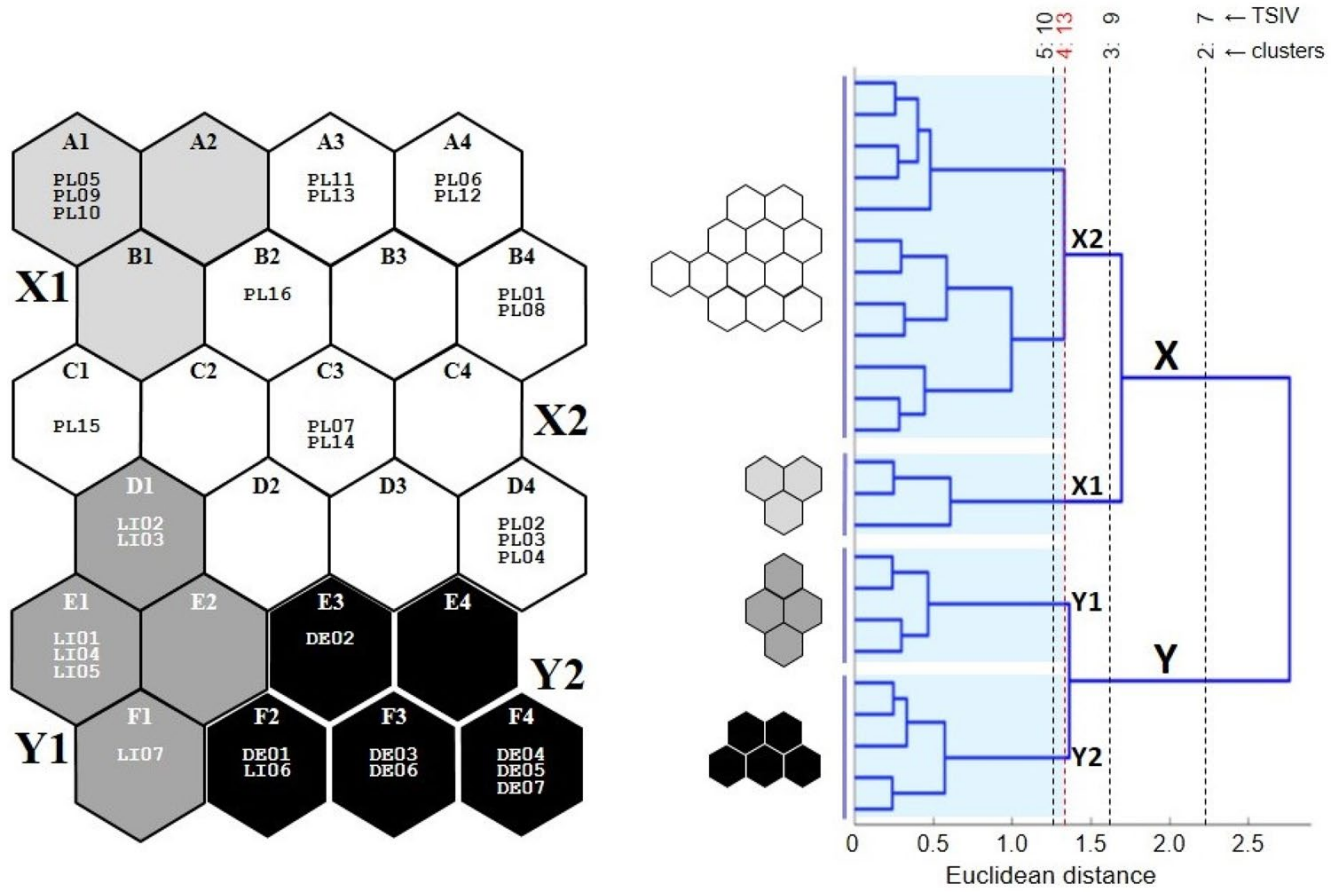
Plots assigned to 24 self-organising map (SOM) output neurons (A1–F4), which were arranged into a two-dimensional lattice (6 × 4). Clusters (X, Y) and subclusters of neurons (X1, X2, Y1 and Y2, which are shown in different degrees of greyness) were identified using hierarchical cluster analysis. The division with the highest number of taxa exhibiting a significant IndVal (TSIV) was chosen and marked in red. Explanation: Germany (DE01–DE07); Lithuania (L01–L07); Poland (PL1: PL01–PL04; PL23: PL05-16 study plots).

**Figure 9:**
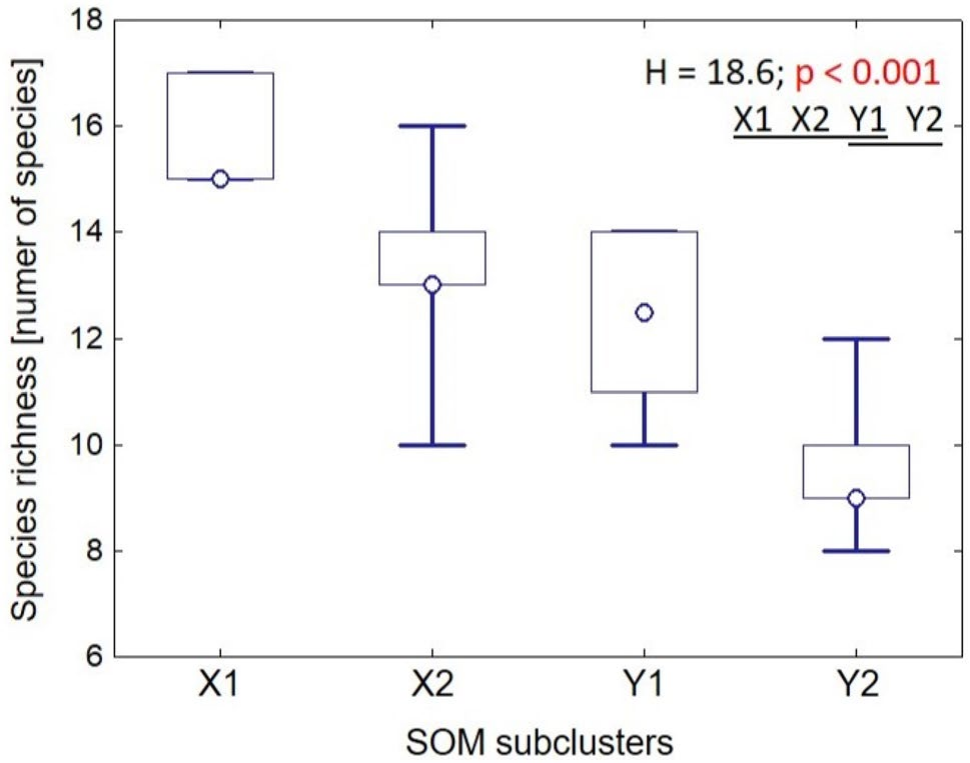
Species richness recorded in plots assigned to SOM subclusters (see Fig. 8). Circle—median, box—interquartile range, whiskers—variability (min–max) range, *H—*Kruskal–Wallis test statistic (d.f. = 3, n_X1_ = 3, n_X2_ = 13, n_Y1_ = 6, n_Y2_ = 8). Subclusters underlined with the same line did not differ in post hoc comparisons.

Two clusters of output neurons, X and Y, were distinguished in the SOM output layer (Fig. 8). Cluster X comprised subclusters X1 (with neurons A1, A2 and B1) and X2 (A3, A4, B2–B4, C1–C4 and D2–D4). Cluster Y contained subclusters Y1 (D1, E1, E2 and F1) and Y2 (E3, E4 and F2–F4) (Fig. 8). Subclusters X1, X2 and Y1 were homogenous in terms of the plot spatial origin. The plots assigned to X1 and X2 were exclusively placed in Poland (PL1, PL23 test points), and those assigned to Y1 in Lithuania (LI); subcluster Y2 contained all plots from Germany (DE), and additionally one from Lithuania (Fig. 8). Y2 (plots mostly from Germany) demsontrated significantly lower species richness than X1 or X2 (plots from Poland) (Fig. 9). The medians for those subclusters were 9, 15 and 13, respectively.

**Figure 10:**
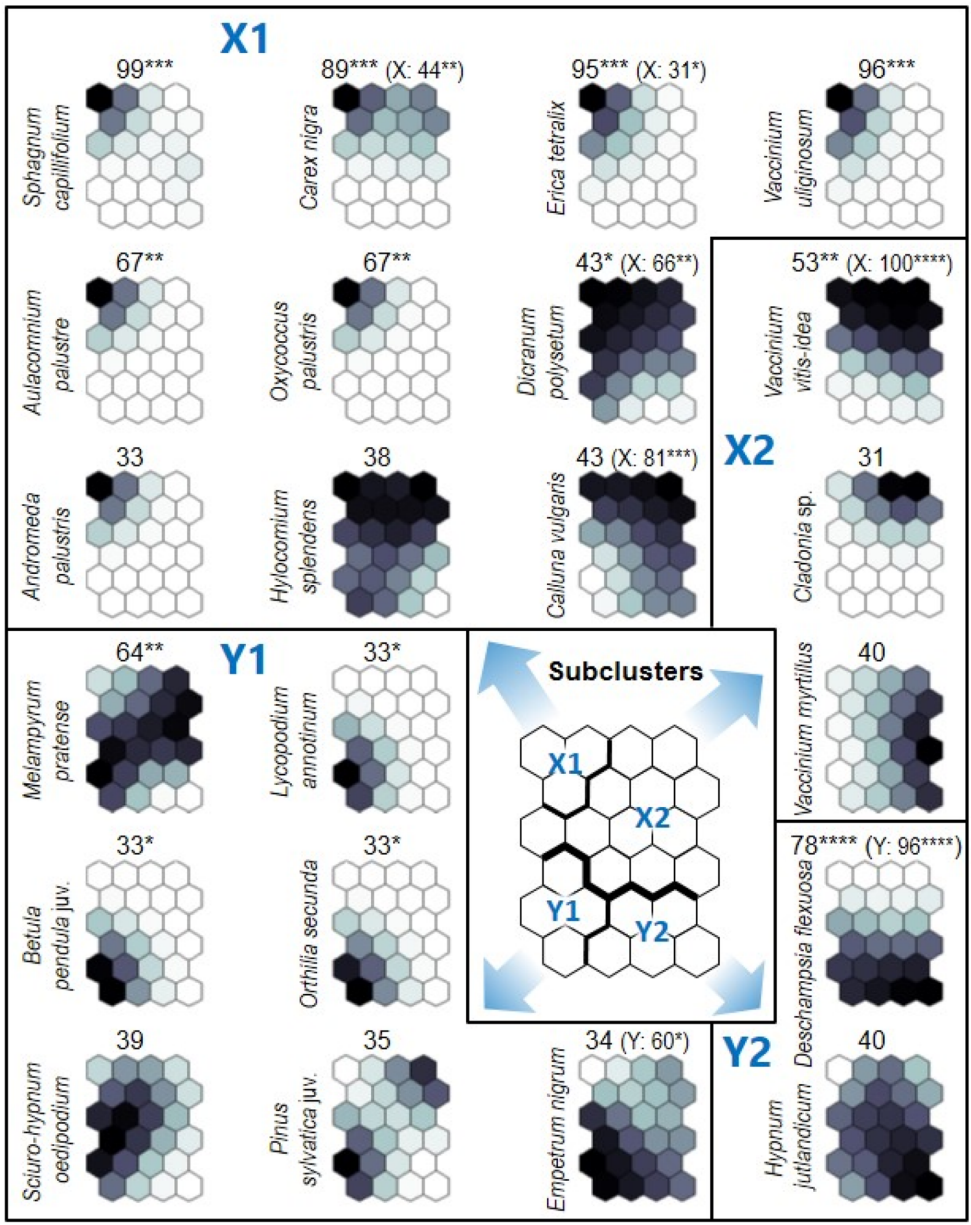
Plant taxa that were associated at *p* ≤ 0.02 with self-organising map (SOM) subclusters X1, X2, Y1 and Y2. The shading (darker for a stronger association in virtual plots) was scaled independently for each taxon. Taxa with similar patterns of greyness usually have similar environmental requirements. The highest indicator value (IndVal; based on real releves) and its significance level (*p ≤ 0.05; **p ≤ 0.01; ***p ≤ 0.001; ****p ≤ 0.0001) in SOM subclusters X1, X2, Y1 and Y2 are presented above each taxon’s plane; additionally, in parentheses the same data are given for SOM clusters X and Y when a given taxon was significantly (*p* ≤ 0.05) associated with them.

The SOM subclusters differed with regard to the number of indicator species, i.e. species that exhibited significantly (*p* ≤ 0.05) higher IndVal values (Fig. 10). The number of indicator species was the highest for X1, i.e. parts of Polish plots. These included *Aulacomnium palustre*, *Carex nigra*, *Dicranum polysetum*, *Erica tetralix*, *Oxycoccus palustris*, *Sphagnum capillifolium* and *Vaccinium uliginosum*. However, the number of indicator species was the lowest for X2, i.e. the remainder of the Polish plots: this included only *Vaccinium vitis*-*idaea*. For Y1 (LI), four such species were recorded: *Betula pendula* young specimens, *Melampyrum pratense*, *Lycopodium annotinum* and *Orthilia secunda* (Fig. 10). Additionally, seven species were significantly associated with clusters: five with X and two with Y. Two of them, *Calluna vulgaris* for X and *Empetrum nigrum* for Y, did not exhibit significant (*p* ≤ 0.05) associations at the subcluster level (Fig. 10).

The conducted research shows that the phytocenosis of Empetro nigri-Pinetum typicum is divided into three variants of the coastal forest: *Calluna–Deschampsia* (from Germany), *Vaccinium vitis–idaea* (from Poland) and *Melampyrum–Deschampsia* (from Lithuania). The floristic differentiation is affected by the climate, and mainly humidity. Thus, the hypothesis "the main driver of forest heterogeneity are climate differences" is also confirmed. Numerical analyses allowed the observed variability to be described, including species pools that discriminate between the described patches. Thus, the next hypothesis "there are indicator species differentiating individual patches" is also confirmed. Principal Component Analysis showed that vascular plants clearly separate the Empetro nigri-Pinetum phytocoenoses; 62% of variability was explained by vascular plants, and only 38% by bryophytes. Thus, the third hypothesis "bryophytes have a higher indicative potential than vascular plants" is not confirmed.

## Discussion

Of the three hypotheses put forward in the introduction, we verified two positively, namely: (H1) the main driver of Empetro-nigri Pinetum heterogeneity are climate differences, and (H2) there are indicator species differentiating individual patches.

The coastal pine forest association on the coast of the southern Baltic Sea was described in several subassociations and several facies (see above). However, the studied plant community, present in protected areas, i.e. the Empetro nigri-Pinetum typicum association, can be classified into three varieties reflected in their geographical distribution and thus climatic conditions: *Calluna–Deschampsia*, found in the west of the southern coast of the Baltic Sea, *Vaccinium vitis–idaea*, found in the center of the coast, and *Melampyrum–Deschampsia*, in the south-eastern part of the coast. The Empetro nigri-Pinetum typicum sub-association is the most widespread and most diverse community among the coastal pine forests on the Polish coast^36^, and the patches studied herein were classified according to the *Empetrum nigrum* facies described by the author (l.c.) and^14^.

The form of *Vaccinium vitis–idaea* offers the highest species richness and occurs on the Polish coast. It is possible to distinguish a variety occurring in wet habitats, which corresponds to the Empetro nigri-Pinetum ericetosum sub-community distinguished by^14^. In addition, patches with higher trophy/anthropogenic disturbance were also documented; for example, the expansion of *Deschampsia flexuosa* and *Vaccinium myrtillus*, and the stronger renewal of pine, have already been described by^36^, documenting patches that are transformed into transitional forms to Betulo-Quercetum.

In contrast, the patches of the community developing on Rügen (DE test point; *Calluna–Deschampsia* form) are poorer in terms of species, with the presence of *Lonicera periclimenum* illustrating the influence of the Atlantic climate. A similar community (i.e. Empetro nigri-Pinetum typicum facies with *Deschampsia flexuosa*) was distinguished by Bosiacka^36^ from the West Pomeranian coast of Poland. The form of *Melampyrum–Deschampsia* from the Curonian Peninsula refers to transitional forest communities in the direction of Betulo-Quercetum, similarly to the disturbed phytocoenoses documented by Bosiacka^36^, and the presence of *Pyrola secunda* and *Lycopodium annotinum* indicates boreal features.

According to^12,14^ the typical sub-association is distinguished by *Listera cordata* and *Ptilium crista–castrensis*. Wojterski (l.c.) notes that both species occurred in almost all patches throughout the unit; however, Bosiacka^36^ report an almost constant absence of these species in the described phytocoenoses, as confirmed by our present findings. Similarly, two other characteristic species, *Goodyera repens* and *Pyrola secunda*, previously reported from the association Empetro nigri-Pinetum typicum, were recorded only sporadically.

Vascular plants distinguish the examined sites much more clearly: 62%, compared to 38% for bryophytes. The study sites were found to be heterogeneous in terms of the dominant species, species composition and the abundance of taxa recorded therein. The performed PCA analysis revealed a clear separation of the plots in terms of quality and quantity. The permanent component is *Empetrum nigrum*, which distinguishes the coastal forest from the fresh forest^3,36^; this taxon also appears to be very strongly influenced by the humidity of the habitat. *Empetrum nigrum* reaches its highest number and percentage share in the eastern patches (325 and 62%, respectively) as does *Melampyrum pratense* (53 and 10%, respectively); in contrast, *Deschampsia flexuosa* is most prevalent in the western patches (354 and 48%). On the other hand, the increase in humidity strongly limits the presence of typical boreal species, including *Vaccinium uliginosum* and *Vaccinium vitis–idaea* e.g.^3,12^.

The SOM and the IndVal index analyses found some species that could be considered distinctive for individual patches. For the Polish plots, these were *Aulacomnium palustre*, *Carex nigra*, *Dicranum polysetum*, *Erica tetralix*, *Oxycoccus palustris*, *Sphagnum capillifolium*, *Vaccinium uliginosum* and *Vaccinium vitis*–*idaea—*their composition reflects the moisture spectrum of the studied phytocenoses^3,36^. In addition, young specimens of *Betula pendula*, *Melampyrum pratense*, *Lycopodium annotinum*, *Orthilia secunda* (LI) and *Deschampsia flexuosa* (DE) were found to be characteristic of other areas.

The third hypothesis namely (H3) bryophytes have a higher indicative potential than vascular plants did not prove to be positively verified in the light of our research.

Bryophytes are valued bioindicators of both the substrates on which they grow and the plant communities in which they develop^8,10,37–43^. However in the present study, neither mosses nor liverworts differentiated the examined sites very clearly; these areas were much better distinguished by vascular plants. This is surprising in the context of generally accepted literature premises. However, the results of the above studies may indicate that bryophytes more easily distinguish different types of forest phytocoenosis, rather than show intraphytocoenotic variability.

The *Dicranum polysetum*, noted in the conducted research, is a typical species from coniferous forests, frequently present throughout the entire ecological amplitude of *Pinus* phytocoenoses^41–45^. However, in Central Europe it is neither characteristic nor distinctive of this type of forest^3^. Due to its commonness in forests, its absence in the area of Empetro nigri-Pinetum typicum in Rügen (DE) is surprising. Another common species from coniferous forests, *Pleurozium schreberi*, was recorded with a quite high frequency throughout the study area. In Central Europe, the species is characteristic of the Vaccinio-Piceetea class and distinctive for the group of lowland oak forests^3^.

In contrast, the most common species in the study area is the moss *Pseudoscleropodium purum*. This species is characteristic of the Empetro nigri-Pinetum association and the coastal variety of the *Vaccinio uliginosi–Pinetum sylvestris* association^3^. In Central Europe, it is also recorded in spruce and fir forests^42^, and less often in pine forests, mixed forests, deciduous forests or in non-forest habitats^44^. *Pseudoscleropodium purum* was recorded in the examined areas with a similar, high frequency, and it appears to constitute a clear, constant share across the entire area of Empetro nigri-Pinetum typicum^36^.

The *Aulacomnium plaustre* moss species is rare in the studied phytocoenosis. In Central Europe, this species is characteristic of the Oxycocco-Sphagnetea class and is distinctive of the Vaccinio uluginosi-Pinetum sylvestris association^3^. However, it was recorded only at two sites in Poland as part of Empetro nigri-Pinetum typicum. Similarly, *Sphagnum capilifolium* was recorded sporadically, at four study plots in Poland. This species is characteristic of the order Sphagnetalia magellanici and the association Sphagnion megellanici, as well as coniferous forests developing on mineral soils e.g. Molinio-Pinetum^3^. *Leucobryum glaucum* moss species was recorded only within the area of Empetro nigri-Pinetum in Poland, and in the literature it is described as characteristic of pine forests developing on mineral soils: Leucobro-Pinetum^3^. The presence of the above-mentioned moss taxa indicates habitat changes (moisture changes) among the patches of the analyzed phytocenosis.

The conducted RDA analyses showed *inter alia* that the number of records of the most common species, *Pseudoscleropodium purum*, is most affected by humidity, with the number increasing with this factor. A literature search reveals that this taxon is indeed associated with coniferous habitats or mixed forests, but with much higher humidity than dry coniferous forests^42–45^.

Several forest taxa of mosses, e.g., *Brachythecium salebrosum*, *Pohlia nutans*, *Polytrichum juniperinum*^42–45^ were recorded sporadically in the study area. However, *Orthodontium lineare*, which is alien to the European bryoflora^44^, was also recorded; this may indicate a disturbance of the examined plots.

Across almost their entire range, but mainly in the western part, coastal pine forests are rapidly degrading^37,46,47^. Well-developed, typical patches with pine are disappearing in favor of grassy facies, and the sites of species reported as characteristic, i.e. *Moneses uniflora* and *Goodyera repens*, are disappearing^12,14,37,46,47^. In view of the above, only patches of the complex located in nature reserves and national parks, i.e. protected habitats (see methods), were included in the present study to examine the relationships between species. In addition, the association is very differently recognized in the phytosociological literature (literature cited above), and even in extreme cases it is not distinguished from the Leucobryo-Pinetum pine forest e.g.^46–48^.

## Summary and conclusions

1. Empetro nigri-Pinetum typicum phytocoenoses are regionally differentiated, and three geographical varieties have been distinguished: *Calluna–Deschampsia* (in Germany), *Vaccinium vitis–idaea* (in Poland) and *Melampyrum–Deschampsia* (in Lithuania). Also, the floristic diversity of Empetro nigri-Pinetum typicum patches is affected by climatic conditions, mainly humidity. Thus, the adopted hypothesis H1 is confirmed.
2. The fact that phytocoenoses from Poland do not show a transitional character between those of Germany and Lithuania indicates the need for caution when formulating assumptions in other studies. Communities located between two points do not have to show an intermediate character.
3. The *Calluna–Deschampsia* (DE) is characterized by the absence of *Dicranum polysetum* and *Melampyrum pratense,* and the maximum occurrence of *Deschampsia flexuosa*. The presence of the latter is a sign of disturbance of the studied phytocenosis. However, the presence of *Lonicera periclimenum* in this area can be interpreted as influence of the Atlantic element.
4. The area of *Vaccinium vitis–idaea* (PL1, PL23) is the most floristically rich and diversified. It is also characterized by the presence of *Erica tetralix*, *Vaccinium vitis*–*idaea* and *Calluna vulgaris*, which are absent in other parts of the studied phytocoenosis.
5. *Melampyrum–Deschampsia* (LI) are characterized by e.g.: *Trientalis europaea*, *Pyrola secunda* and *Lycopodium annotinum*. This can be interpreted as the influence of the boreal element.
6. Numerical analyzes allowed the species pools that discriminate between the described patches to be demonstrated. As the result, the hypothesis H2 is confirmed.
7. Vascular plants much clearer differentiate the examined study plots (62%) than bryophytes (38%)-the hypothesis H3 is not confirmed.
8. The IndVal index revealed the following species as indicators for individual phytocoenoses: *Deschampsia flexuosa* for *Calluna–Deschampsia* group; *Aulacomnium palustre*, *Calluna vulgaris*, *Carex nigra*, *Dicranum polysetum*, *Erica tetralix*, *Oxycoccus palustris*, *Sphagnum capillifolium*, *Vaccinium uliginosum* and *Vaccinium vitis*–*idaea* for *Vaccinium vitis–idaea* group; and young specimens of *Betula pendula*, *Lycopodium annotinum*, *Melampyrum pratense* and *Orthilia secunda* for *Melampyrum–Deschampsia* group.

### Supplementary Information

Supplementary Information.

Supplementary Figure 1.

Supplementary Tables.

## Data Availability

All data generated or analysed during this study are included in this published article and its supplementary information files.
