# Supplementary material for: Vascular plants and mosses as bioindicators of variability of the coastal pine forest (Empetro nigri-Pinetum)
Source: Sci Rep. 2024 Jan 2;14:76. doi: 10.1038/s41598-023-50189-y (PMC10761821; doi:10.1038/s41598-023-50189-y)
Supplement: Supplementary file 2 — Supplementary Figure 1. [file 41598_2023_50189_MOESM2_ESM.docx]

Supplementary figure 1


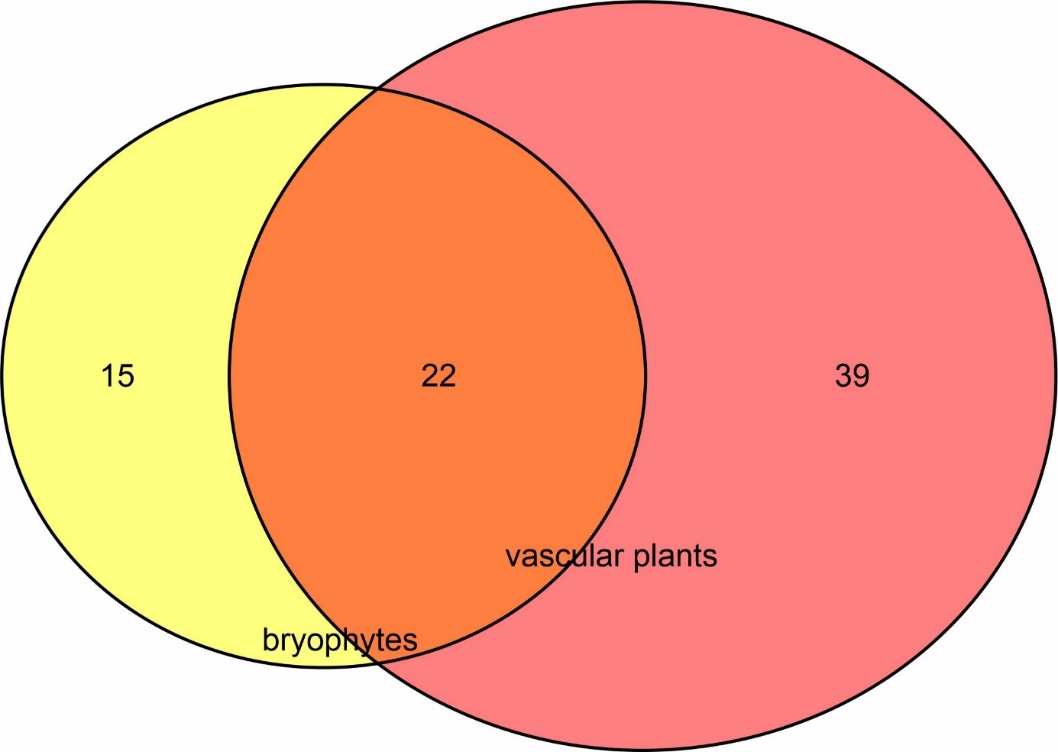


Fig. Venn diagram of variance partioning of data grouped into two subsets: bryophyte and vascular plant species. The total R2 involving all predictors (10 vascular plant and 6 bryophyte species) amounted to 0.993. The forest stands variation based on the first six PCA axes, which describe 77.9% of their total variability.
