# Supplementary material for: Vascular plants and mosses as bioindicators of variability of the coastal pine forest (Empetro nigri-Pinetum)
Source: Sci Rep. 2024 Jan 2;14:76. doi: 10.1038/s41598-023-50189-y (PMC10761821; doi:10.1038/s41598-023-50189-y)
Supplement: Supplementary file 3 — Supplementary Tables. [file 41598_2023_50189_MOESM3_ESM.docx]

Supplementary table 1. Diversity measures for individual areas - bryophytes.

|  | DE | PL1 | PL23 | LI |
| --- | --- | --- | --- | --- |
| Taxa_S | 6 | 7 | 7 | 6 |
| Individuals | 597 | 316 | 1269 | 734 |
| Dominance_D | 0,3581 | 0,287 | 0,2458 | 0,2958 |
| Simpson_1-D | 0,6419 | 0,713 | 0,7542 | 0,7042 |
| Shannon_H | 1,255 | 1,52 | 1,615 | 1,455 |

Supplementary table 2. Statistical significance of differences between individual areas in terms of species occurrence. PERMANOVA permutation analysis of variance was used - bryophytes.

|  | SS | df | MS | F | pseudo-F | p |
| --- | --- | --- | --- | --- | --- | --- |
| Between-group sum of squares | 2971 | 3 | 990,3333 | 3,331 | 3,327 | <0,01 |
| Within-group sum of squares: | 7729 | 26 | 297,2692 |  |  |  |
| Total sum of squares: | 10700 | 29 |  |  |  |  |

Results of the post-hoc test

|  | DE | PL1 | PL23 | LI |
| --- | --- | --- | --- | --- |
| DE |  | 0,0764 | 0,0001 | 0,0068 |
| PL1 | 0,0764 |  | 0,0775 | 0,0691 |
| PL23 | 0,0001 | 0,0775 |  | 0,3704 |
| LI | 0,0068 | 0,0691 | 0,3704 |  |

Supplementary table 3. Diversity measures for individual areas - vascular plants.

|  | DE | PL1 | PL23 | LI |
| --- | --- | --- | --- | --- |
| Taxa_S | 5 | 8 | 12 | 9 |
| Individuals | 5202 | 2543 | 7557 | 3693 |
| Dominance_D | 0,36 | 0,2185 | 0,2729 | 0,4158 |
| Simpson_1-D | 0,64 | 0,7815 | 0,7271 | 0,5842 |
| Shannon_H | 1,212 | 1,702 | 1,555 | 1,301 |

Supplementary table 4. Statistical significance of differences between individual areas in terms of species occurrence. Permutation analysis of variance PERMANOVA – vascular plants was used.

|  | SS | df | MS | F | pseudo-F | p |
| --- | --- | --- | --- | --- | --- | --- |
| Between-group sum of squares | 1215700 | 3 | 405233,3 | 12,766 | 12,77 | <0,01 |
| Within-group sum of squares: | 825300 | 26 | 31742,31 |  |  |  |
| Total sum of squares: | 2041000 | 29 |  |  |  |  |

Results of the post-hoc test

|  | DE | PL1 | PL23 | LI |
| --- | --- | --- | --- | --- |
| DE |  | 0,0062 | 0,0001 | 0,0008 |
| PL1 | 0,0062 |  | 0,0004 | 0,0034 |
| PL23 | 0,0001 | 0,0004 |  | 0,0001 |
| LI | 0,0008 | 0,0034 | 0,0001 |  |

Supplementary table 5. Permutation test (RDA, Canoco) of species occurred in Empetro-nigri association in the Baltic region.

| Name | Explains % | pseudo-F | P | P(adj) |
| --- | --- | --- | --- | --- |
| *Empetrum nigrum* Group | | | | |
| *Emp nig* | <0,1 | 4,3 | 0,162 | 0,321 |
| *Sci oed* | <0,1 | 2,6 | 0,128 | 0,303 |
| *Lyc ann* | <0,1 | 2,4 | 0,130 | 0,303 |
| *Ver cha* | 0,2 | 3,6 | 0,040 | 0,140 |
| *Pse pur* | 0,1 | 3,3 | 0,072 | 0,189 |
| *Ple sch* | <0,1 | 1,1 | 0,326 | 0,507 |
| *Bet juv* | <0,1 | 2,0 | 0,154 | 0,321 |
| *Erica tetralix* Group | | | | |
| *Oxy pal* | 1,1 | 6,7 | 0,006 | 0,042 |
| *Dic pol* | 0,7 | 6,4 | 0,006 | 0,042 |
| *Eri tet* | 2,2 | 6,0 | 0,024 | 0,092 |
| *Pti cil* | 0,2 | 3,3 | 0,054 | 0,162 |
| *Pol jun* | 0,1 | 3,1 | 0,072 | 0,189 |
| *Hyl spl* | <0,1 | 1,9 | 0,156 | 0,321 |
| Other | | | | |
| *Vac vit* | 65,6 | 53,4 | 0,002 | 0,028 |
| *Des fle* | 15,1 | 21,2 | 0,002 | 0,028 |
| *Cal vul* | 7,9 | 18,1 | 0,002 | 0,028 |
| *Hyp jut* | 0,9 | 7,1 | 0,004 | 0,042 |
| *Lon per* | 2,1 | 7,3 | 0,018 | 0,076 |
| *Leu gla* | 0,5 | 6,9 | 0,012 | 0,063 |
| *Ort lin* | 1,6 | 6,8 | 0,012 | 0,063 |
